# Self-Microemulsifying Drug Delivery Systems: An Attractive Strategy for Enhanced Therapeutic Profile

**DOI:** 10.1155/2014/964051

**Published:** 2014-12-08

**Authors:** Samatha Akula, Aravind Kumar Gurram, Srinivas Reddy Devireddy

**Affiliations:** ^1^Vaageswari College of Pharmacy, Karimnagar, Telangana 505001, India; ^2^Manipal College of Pharmaceutical Sciences, Manipal, Karnataka 576104, India

## Abstract

Ease of administration and painless approach made oral route the most preferred. Poor oral bioavailability is pronounced with the majority of recent active ingredients because of dissolution rate limited absorption. Failure to attain intended therapeutic effect of the poor water soluble drugs by this route led to development of novel drug delivery systems which will fulfill therapeutic needs with minimum dose. Although many formulation approaches like solid dispersions, complexation, pH modification, and cocrystals exist, lipid based delivery systems finding increased appliance with the apparent increase in absorption of drug. Among lipid based formulations, self-microemulsifying formulations (droplet size < 100 nm) are evident to improve the oral bioavailability of hydrophobic drugs primarily due to their efficiency in facilitating solubilization and in presenting the hydrophobic drug in solubilized form whereby dissolution process can be circumvented. Various components that are used to formulate these dosage forms like surfactants and lipids contribute to the overall improvement in oral bioavailability via promoting the lymphatic transport; thereby hepatic first pass metabolism can be surmounted. The present paper gives exhaustive information on the formulation design and characterization of SMEDDS along with the probable mechanisms by which the bioavailability can be improved with SMEDDS.

## 1. Introduction

Advances in* in vitro* screening methods like combinatorial chemistry are leading to the emergence of many potential chemical components with marked therapeutic activity. However, about 40% of them are poor water soluble drugs due to which their absorption is dissolution rate limited and have low oral bioavailability [1]. Apart from this, oral delivery of these drugs suffers from drawbacks like rapid metabolism, lack of constant drug level in blood/plasma, and interindividual variability [2]. The inherent properties of chemical moieties can be modified by various means like particle size reduction and by salt formation of the drug to improve the bioavailability without any formulation approach. However it is not always possible with all drug molecules. Salt formation is not achievable with neutral drug molecules and the performance of salt forms in the GIT may not be efficacious because they will not exist in the original form; instead they get converted into acid or base by which the approach may not be useful. Particle size reduction may not be useful in case of all drug components because of disadvantages associated with fine powders like poor wettability and low stability [3]. Oral delivery of poor water soluble drugs using lipid as vehicle is a new and recent approach to overcome the aforesaid problems. Formulation excipients like surfactants used in the lipid based formulations will aid in achieving the goal [2]. The evident increase in bioavailability of poor water soluble drug with fatty meal is base line in the design of lipid based formulations [4–6] and this was investigated and proved in case of many drugs like griseofulvin, halofantrine, danazol, atovaquone, and troglitazone [2]. Coadministration of lipid with the lipophilic drug is as advantageous because it contributes to the enhancement of bioavailability of the drug by the following mechanisms.

Stimulation of body secretions that help in digestion of lipids: administration of lipid can stimulate the biliary and pancreatic secretions which are helpful for the digestion of lipids. The enzymes present in the secretions are water soluble and act at water/lipid interface. Fatty acids liberated from the lipid digestion process interact with the bile salts and result in the formation of mixed micelles and micelles in which the drug gets solubilized.

Prolongation of GI residence time: administration of lipid along with the drug allows the drug to be present for prolonged duration of period in the GIT which facilitates the absorption of the drug [2].

Stimulation of lymphatic transport: the highly lipophilic drug (log *P* > 5) which has high solubility in triglycerides (>50 mg/mL) can undergo lymphatic transport when coadministered with esters of unsaturated long chain fatty acids; thereby bioavailability can be improved [7–9]. This restricted lymphatic transport is mainly due to low lymph-to-blood flow ratio. This enhanced lymph delivery of the drug can bypass the first pass extraction whereby the bioavailability of drugs that undergo extensive first pass effect can be improved. Lipid based delivery system like lopinavir loaded SLN was found to deliver high amount of drug in to lymphatic circulation compared to control (pure drug) due to which the first pass extraction of this drug was inhibited and hence bioavailability was found improved [10].

Increased intestinal wall permeability: opening of tight junctions in the intestine caused by lipids contributes to the increased permeability of poorly permeable drugs [7]. Although this mechanism is not essential in case of BCS Class II drugs, it leads to marked improvement in absorption of Class IV drugs which have both dissolution and permeability rate limited absorption.

Reduced efflux of the drug in the GIT: lipids such as anionic phospholipids (cardiolipin and phosphatidylserine) may inhibit permeability glycoprotein (P-gp) by interaction with membrane lipids. So the drugs which have propensity to be effluxed from the GIT can be formulated as lipid based delivery systems for the improvement of bioavailability [11]. The inhibitory effect is due to competition for binding with the transporter and due to membrane perturbation caused by the excipients, mainly surfactants. The residence time of the drug can be prolonged by this inhibition of efflux [8].

Among various lipid based formulations (liposomes, solid lipid nanoparticles, self-dispersing tablets, and solid solutions), self-microemulsifying formulations are receiving more attention by formulation scientists as these are advantageous in the aspect of their stability, self-dispersing nature, ease of preparation, and scale-up. SMEDDS are the isotropic, clear mixtures of oils and surfactants and sometimes include cosolvents/cosurfactants. These are designed to form O/W microemulsions with mild agitation produced by the motility of GIT followed by solubilization and absorption of drug. SMEDDS usually produce microemulsions of droplet size below 100 nm upon dilution [3].

## 2. Importance of SMEDDS

SMEDDS offer the following advantages.

- Irritation caused by prolonged contact between the drug and the wall of the GIT can be surmounted by the formulation of SMEDDS as the microscopic droplets formed help in the wide distribution of the drug along the GIT and these are transported quickly from the stomach [8].
- Upon dispersion in water, these formulations produce fine droplets with enormous interfacial area due to which the easy partition of the drug from the oil phase into the aqueous phase is possible which cannot be expected in case of oily solutions of lipophilic drugs [8].
- SMEDDS are advantageous over emulsions in terms of the stability because of the low energy consumption and the manufacturing process does not include critical steps. Simple mixing equipment is enough to formulate SMEDDS and time required for preparation is also less compared to emulsions [3, 12].
- Poor water soluble drugs which have dissolution rate limited absorption can be absorbed efficiently by the formulation of SMEDDS with consequent stable plasma-time profile [3]. Constant plasma levels of drug might be due to presentation of the poorly soluble drug in dissolved form that bypasses the critical step in drug absorption, that is, dissolution [13].
- Along with the lipids, surfactants that are commonly used in the formulation of SMEDDS like Tween 80, Spans, Cremophors (EL and RH40), and Pluronics are reported to have inhibitory action on efflux transporters which help in improving bioavailability of the drugs which are substrates to the efflux pumps [11, 13–15]. Surfactant named d-a-tocopheryl polyethylene glycol 1000 succinate (TPGS) produced by esterification of vitamin E succinate and polyethylene glycol 1000 was proved to have inhibitory effect on efflux transporters like P-glycoprotein [16]. The efflux of paclitaxel from the GIT was found to be inhibited with formulation prepared using surfactant named polysorbate 80 [17].
- Drugs which have propensity to be degraded by the chemical and enzymatic means in GIT can be protected by the formulation of SMEDDS as the drug will be presented to the body in oil droplets [3].
- Microemulsion preconcentrate is advantageous over microemulsion to dispense in the form of liquid filled soft gelatin capsules [18].
- SMEDDS are advantageous over SEDDS as the former are less dependent on bile salts for the formation of droplets by which better absorption of the drug is expected compared to SEDDS [19].
- Surfactants of high HLB like Tween 80 are reported to increase the permeability of the drug when administered along with the formulation due to the loosening effect of these on tight junctions [20].

## 3. Composition of SMEDDS

### 3.1. Lipid (Oils)

Oils are the important component of SMEDDS, as solubilization and access of the drug to the lymphatic circulation of poor water soluble drugs depend on the type and concentration of oil used for formulation. Digestive lipids such as triglycerides, diglycerides, fatty acids, phospholipids, cholesterol [1], and other lipids based on synthetic origin offer improvement in bioavailability of the drug in contrast to the nondigestible lipids with which reduced bioavailability may occur due to impairment in absorption caused by retention of the fraction of administered drug in the formulation itself.

Although edible oils based on natural origin are favored, they are not useful as they do not have sufficient capacity to solubilize large amount of lipophilic drug and self-emulsification is also problematic with them as they possess a large molecular volume [21]. Instead, modified or hydrolyzed oils of vegetable origin are beneficial due to their superior emulsification properties and compatibility with oral administration as their end products of degradation bear a resemblance to the end products produced by digestion process in the intestine [22]. Polyglycolized glycerides of varying HLB attributed to difference in fatty acid chain length and PEG chain length are used along with vegetable oils for the improvement in the bioavailability of drug and are used for the reason of better tolerability by the human body [12]. Triglycerides with long and medium chain length containing different degrees of saturation are commonly used in the preparation of SMEDDS [3]. Medium chain triglycerides have the capacity to get digested efficiently compared to the long chain triglycerides [12, 23] and also exhibit greater fluidity, improved solubility properties, and good ability to self-emulsify along with the reduced tendency towards oxidation due to which they contribute to the increase of drug absorption and in turn have positive effects on bioavailability. These attractive properties made them more commonly used compared to LCTs [12, 24]. Prajapati et al. performed a study for microemulsion area in phase diagram and concluded that the mixture of lipids (medium chain fatty acids) composed of monoglyceride : diglyceride or triglyceride in 1 : 1 ratio produced expanded microemulsion phase and reduced gel phase which is suitable for oral administration [25]. Semisynthetic medium chain derivatives are superior to MCTs for the reason that they are amphiphilic in nature with surfactant properties [3, 22].

Though medium chain triglycerides have superior properties to long chain triglycerides, the drug access to lymph is not possible with them and it is possible only with lipids composed of LCTs. Oils like cottonseed oil and soybean oil composed of LCTs are reported to enhance the bioavailability of highly lipophilic drugs by stimulation of lymphatic transport of drugs [7, 26]. Mepitiostane (prodrug of epitiostanol) and Mepitiostaneolefin with octanol : water partition coefficients of 6 and 5.1 were proved to undergo significant lymphatic transport when given along with lipids like long chain triglycerides [27]. Not only the type of lipid but also the concentration of lipid has effect on drug transfer into lymphatics and this was investigated with sirolimus SMEDDS where enhanced lymphatic transfer of drug was achieved with formulation containing ≥25% of oil content [28]. The lipids with high unsaturation have the tendency to get oxidized and the resultant peroxide may lead to detrimental effect on drug release due to the delay in capsule disintegration. This problem can be addressed by various means like including antioxidants in the formulation, by controlling the utilization of highly unsaturated lipids and by employing sealed hard gelatin capsules that possess impermeability to oxygen [29].

### 3.2. Surfactants

A surfactant is needed to adopt self-emulsification property by SMEDDS which is prime process to form microemulsion and it is also helpful to solubilize the hydrophobic drug; in turn the dissolution rate can be improved. The solubilization behavior of surfactant for the drug gained popularity due to its inhibitory effect on drug precipitation* in vivo *[30]. Permeability barrier that is intestinal cell membrane comprised of lipids can be disrupted by surfactant partition; thereby permeability will be enhanced [3]. The opening of tight junctions by the surfactants also contributes to the improvement in permeability and this was explored with the study conducted by Sha et al. where enhanced permeability of the drug was observed with surfactant labrasol due to opening of tight junctions [31]. The inhibitory effect of surfactants on p-glycoprotein helps in the improvement of overall bioavailability of many drugs that are substrates to p-glycoprotein transporter [13].

Although natural surfactants are less toxic, the efficiency of self-emulsification is limited [3]. For spontaneous emulsification, the surfactants are required to be selected with attention to attain ultralow interfacial tension [32]. The selection of surfactant is based on HLB value. The surfactants with high HLB facilitate the formation of O/W microemulsion [30]. Surfactants with hydrophilic nature, that is, HLB value of greater than 12, along with water soluble cosolvents, are used for drugs with relatively low octanol : water partition coefficient to increase the solvent capacity of the formulation and these systems produce very fine droplets of size less than 100 nm with high surfactant concentration [33]. The less toxicity offered by nonionic surfactants like oleates, polysorbates, polyoxyls, and so forth compared to ionic surfactants allows them to be used more commonly in the formulation of SMEDDS [29]. With commonly used lipids in the formulation of SMEDDS like medium and long chain triglycerides, the nonionic surfactants like oleates of HLB 11 having unsaturated acyl side chains are more suitable excipients for efficient self-emulsification [24].

Most of the surfactants have impact on lipid digestion that is catalyzed by lipase in various ways like the formation of complexes with the enzyme at interface, by preventing the adsorption of enzyme at interface or by the interaction with the lipase itself. Inhibition of lipid digestion may also occur as the surfactant has the tendency to interact with other components like bile salts and phospholipids. When different surfactants are compared in this aspect, little impact on lipid digestion is observed in case of nonionic surfactants, promoting effects on lipid digestion with the use of cationic surfactants and inhibitory effects with anionic surfactants [34].

Utility range of surfactants for the formation of stable SMEDDS is about 30–60%.

Care should be exercised to minimize the concentration of surfactant as minimum as possible because the use of high concentration of surfactants has disadvantages like GI irritation, [3] decrease in self-emulsification efficiency, and dehydrating effect on soft and hard gelatin capsules (caused by some of the nonionic surfactants like polysorbates and polyoxyls) with consequent brittleness [29]. At high concentrations of surfactant, GI irritation occurs due to tissue damage [35] and the efficiency of self-emulsification capacity decreases which may be due to the formation of liquid crystalline phase at the interface which in turn is due to viscous nature [33]. Although there is an indirect relationship between droplet size and surfactant concentration, it exists only to about a certain range due to stabilization effect caused by surfactant on oil droplets by its accumulation at oil/water interface. Above the range, the opposite effect is observed due to the disruption of interface with the surfactant of high concentration that leads to entry of water into oil droplets [3].

### 3.3. Cosolvent

Cosolvents facilitate the dissolution of surfactant and hydrophobic drug in oil phase because of their ability to access the entry of water into the formulation. These excipients play the role of cosurfactant in microemulsion system [3, 30]. Some of the commonly used cosolvents are short chain alcohols like ethanol, n-butanol, propylene glycol, and polyethylene glycol [3, 30]. The addition of cosolvents such as short chain alcohols imparts flexibility to the interface that is helpful for the free movement of the hydrophobic tails of surfactant at interface which in turn imparts dynamic behavior to microemulsions [32]. Alcoholic, low molecular weight cosolvents may cause precipitation of the drug when the formulation is filled in gelatin capsules since they are absorbed onto the capsule shells [24]. Along with the nature, the concentration of cosurfactant also has an impact on drug precipitation.

Due to their high polarity, they tend to migrate towards aqueous phase upon dispersion into aqueous media leading to drug precipitation. Hence it is advisable to formulate SMEDDS in minimum concentration [36]. The selection of suitable surfactant and cosurfactant should be done by considering the efficacy, irritancy, change in efficacy caused by repeated administration of formulation, their interaction with the proteins and lipids of the mucosa, and metabolic pathway followed by them [22].

### 3.4. Drug

When poor solubility is the major reason for insufficient absorption of drug, lipid based formulations are preferred [29]. Apart from poor water solubility, appreciable solubility of the drug in oil phase is important in the selection of suitable drug candidate for the formulation of lipid based delivery systems like SMEDDS [24]. The drug should be sufficiently hydrophobic to be soluble in the lipid component of the formulation; that is, octanol : water partition coefficient should be high (log *P* > 5) to incorporate the whole required dose of the drug in one dosage unit [24, 29]. Most of the hydrophobic drugs have good solubility in synthetic oils and surfactants compared to that in oils from natural source [22]. The greater bioavailability from the SMEDDS can be achieved when the dose is very low especially for the drugs with high octanol : water partition coefficient. The absorption of the drug from SMEDDS is primarily dependent on its solubility in water and lipid phase [33]. Drugs that have poor bioavailability because of presystemic metabolism can be formulated as SMEDDS provided that the drug should have high solubility in long chain triglycerides (>50 mg/mL) and octanol : water partition coefficient of greater than five [9].

## 4. Mechanism of Self-Emulsification

The free energy of the emulsion can be described by the following equation:

(1) $$\begin{array}{c} \Delta G=\sum N\Pi r2\sigma . \end{array}$$

Δ*G*  is the free energy, *N* is the number of droplets, *r* is the radius of droplets, and *σ* is the interfacial energy.

From this equation, it is evident that the lower the interfacial energy the lower the free energy.

Self-emulsification occurs when the energy involvement in the dispersion is greater than the energy required for the formation of droplets [22].

The free energy of conventional emulsion is very high as high energy is required to form new surface between two immiscible phases like oil and water. Due to high free energy, the emulsion may not be stable and the two phases tend to separate. But in case of SMEDDS, emulsion formation occurs instantaneously because the free energy of the system is very low and sometimes negative due to the presence of flexible interface. On mixing oil and surfactant/cosurfactant mixture with water, up on mild agitation, an interface is formed between two phases. Then, aqueous phase penetrates through interface and gets solubilized within the oil phase up to the solubilization limit. Increased water penetration causes the formation of dispersed liquid crystalline phase. The amount of liquid crystalline phase depends on the surfactant concentration. Upon mild agitation of SMEDDS, water penetration occurs rapidly and leads to the disruption of interface and droplets will be formed [3]. As microemulsions are thermodynamically stable, equilibrium exists within the system although there is continuous exchange of matter between the different phases. Exchange of matter usually occurs in two different ways like fusion of small droplets followed by the fission of larger droplet into small droplets and fragmentation of droplets which later coagulate with other droplets [32].

## 5. Effect of Drug Addition on SMEDDS

Optimal drug incorporation can be achieved if good compatibility exists between the added drug and the system with respect to physical and chemical properties. The drug may cause changes in the behavior of the system by reacting with the formulation components or by entering into the interfacial surface where surfactant molecules exist. This problem is more pronounced in case of SMEDDS where the droplet size is much smaller than other self-emulsifying formulations. Preformulation studies like determination of solubility of drug in various components of formulation and construction of phase diagram to know the exact emulsification area can help in resolving the problem of unwanted effects of drug incorporation on optimal SMEDDS [3].

The drug loading also has influence on the droplet size. Bandivadeka et al. studied the effect of drug addition on droplet size and concluded that increased amount of drug addition leads to the increase in particle size and this may be due to the decreased availability of surfactant to reduce the particle size [37]. If the drug has propensity to form H-bonds with ethoxy chains of surfactant, it can affect the performance of SMEDDS. If the drug is highly lipophilic and does not have the ability to form H-bonds, there will not be any effect of drug addition even in high concentrations. The construction of phase diagrams in the presence of drug is helpful for the determination of effect of drug addition on the existence of microemulsion area [24].

## 6. Formulation Design

Formulation of SMEDDS involves the following steps.

1. Screening of excipients.
2. Construction of pseudoternary phase diagram.
3. Preparation of SMEDDS.
4. Characterization of SMEDDS.

### 6.1. Screening of Excipients

#### 6.1.1. Solubility Studies

These are mainly useful for the selection of the most suitable excipients that can be used in the preparation of SMEDDS and helps in the prediction of drug precipitation* in vivo *[33]. Solubility of the drug in various oils, surfactants, and cosurfactants should be tested [38, 39]. These studies are generally performed by shake flask method in which the drug is usually added to the excipient in excess amount and then shaken for 48 hours in water bath shaker or in air oscillator at room temperature [39]. Then, the samples should be subjected to centrifugation followed by filtration through 0.45 *μ*m filters and drug content should be determined. These solubility studies are generally performed with the objective of choosing oil that shows maximum solubility for the drug and surfactant/cosurfactant which have maximum capacity to solubilize the drug [40]. The other objective is achievement of optimum drug loading with minimized total volume of the formulation. Drug precipitation may occur from diluted SMEDDS which is dependent on octanol :  water partition coefficient of the drug and also on the level of involvement of surfactant in the solubilization of the drug.

#### 6.1.2. Screening of Surfactants and Cosurfactants for Their Self-Emulsification Ability

The emulsification ability of surfactants can be known by mixing the equal proportions of selected oil and surfactant which is followed by homogenization. When this mixture is added to the double distilled water, the number of flask inversions required to form homogenous emulsion is noted and this gives indication about ease of emulsification. Then, the resultant microemulsion should be tested for clarity, turbidity, and percentage transmittance. The surfactants that show highest emulsification efficiency, that is, that show high percentage transmittance and that require low flask inversions, should be selected [41, 42]. Similarly, the cosurfactants should be screened with the same procedure by mixing selected surfactant and oil phase with cosurfactant [42].

### 6.2. Construction of Pseudoternary Phase Diagram

These are the diagrams which represent change in phase behavior of the system according to the change in composition. Ternary phase diagram is used to study the phase behavior of three components. In SEDDS, this represents the system with three components like oil, water, and surfactant. But in case of SMEDDS, the additional component like cosurfactant/cosolvent addition is most common. Ternary diagram contains three corners that correspond to the 100% of the particular component. In case of addition of fourth component, the ternary diagram can be called pseudoternary phase diagram as one of the corners corresponds to the mixture of two components like surfactant and cosurfactant [43].

For construction of pseudoternary phase diagram, mixtures containing different compositions of microemulsion components should be evaluated for emulsification efficiency [44]. At different compositions, different structures may be formed like emulsions, microemulsions, micelles, inverted micellar forms, and so forth and the extent of formation of these structures can be known with the construction of phase diagram. This phase diagram helps in the determination of dilutability of formulation and in getting information about the different compositions that form monophasic clear solutions [13]. Pseudoternary diagrams are constructed by keeping the ratio of any two of the four components as constant and this ratio along with the remaining two components generally forms three corners of the phase diagram. This fixed (mixture) ratio is generally formed by the combination of surfactant and cosurfactant [40, 45] and sometimes it may be the mixture of oil and surfactant [12]. This is mixed with the required volume of the third phase like oil [45, 46] or cosurfactant [12]; then the other component which is usually water is added in incremental amounts and for every addition of fourth component, the solution should be tested for the clarity, flowability, time for self-emulsification, and dispersibility [40]. The total percent concentration of all components in each mixture should be 100% [44].

Then pseudoternary diagram should be plotted with the help of suitable software. The samples which formed clear solution should be denoted by suitable symbols in the phase diagram [47]. The area that is formed when these points are joined indicates the monophasic microemulsion existing area [48] and wide area indicates the good emulsification efficiency [40].

#### 6.2.1. How to Read a Typical Ternary Diagram (Figure 1)

The following points may be useful to read and to understand ternary diagram in an easy way.

The three corners of the typical ternary diagram represent three components, that is, A, B, and C. The arrow towards BA indicates increase in proportion of A from 0% concentration (at point B) to 100% concentration (at point A), the arrow towards AC indicates the increase in proportion of C from 0% concentration (at point A) to 100% concentration (at point C), and similarly the arrow towards CB indicates the increase in proportion of B from 0% concentration (at point C) to 100% concentration (at point B).

Composition at point “O” can be known by the following.

- Draw a line that is parallel to CB from point O towards AB. The point where this line intersects with AB indicates the percent composition of A at point O (X).
- Then, percent composition of B at point O can be known by drawing a line that is parallel to AC towards BC. The point where this line intersects with BC indicates the percent composition of B at point O (Y).
- Similarly, the percent composition of C at point O can be known by drawing a line that is parallel to AB towards AC (Z).

### 6.3. Preparation of SMEDDS

The preparation involves the addition of drug to the mixture of oil, surfactant, and cosurfactant and then it should be subjected to vortexing [49]. In some cases, drug is dissolved in any one of the excipients and the remaining excipients are added to the drug solution [46]. Then, the solution should be properly mixed and tested for the signs of turbidity. After equilibration at ambient temperature for 48 hours, the solution should be heated for the formation of clear solution, if required. Depending on the final volume, the formulation should be stored in capsules of suitable size [39].

### 6.4. Characterization of SMEDDS

#### 6.4.1. Visual Evaluation

The assessment of self-emulsification is possible by visual evaluation. After dilution of SMEDDS with water, the opaque and milky white appearance indicates the formation of macroemulsion whereas the clear, isotropic, transparent solution indicates the formation of microemulsion [3, 30, 50]. Assessment of precipitation of drug in diluted SMEDDS is also possible by visual evaluation. The formulations can be considered as stable when drug precipitation is not evident. Precipitation is common if the formulation contains water soluble cosolvents and can be avoided by increasing the concentration of surfactant [51].

#### 6.4.2. Droplet Size Analysis

The droplet size is mainly dependent on the nature and concentration of surfactant [35]. Microemulsion formed upon dilution with water produces droplets of very narrow size and size distribution for effective drug release,* in vivo *absorption, and also stability. Spectroscopic techniques like photon correlation spectroscopy and microscopic techniques are used for droplet size analysis [3, 30]. Dynamic light scattering techniques employing Zetasizer can also be used for droplet size analysis [52]. Samples should be diluted suitably before analyzing for size evaluation [38, 39, 49]. The determination of polydispersity index (PDI) gives suitable information about size distribution. The low value of PDI indicates the uniform and narrow size distribution [53].

#### 6.4.3. Zeta Potential Measurement

Zeta potential is generally measured by zeta potential analyzer [49] or zeta meter system [54]. Value of zeta potential indicates the stability of emulsion after appropriate dilution. Higher zeta potential indicates the good stability of formulation [54]. Usually the value of zeta potential is negative due to the presence of free fatty acids [30] but when cationic lipid such as oleylamine is used, the positive charge gets developed [3]. The droplets of positive charge have the property of interacting efficiently with the mucosal surface of the GIT and these interactions are of electrostatic nature due to which strong adhesion can be expected with increased absorption [8].

#### 6.4.4. Time for Emulsification

The time needed for self-emulsification for different formulations can be assessed generally using dissolution apparatus USP type II in which the formulation is added dropwise to the basket containing water and observing the formation of clear solution under agitation provided by paddle at 50 rpm [49]. Assessment of self-emulsification helps to determine the efficiency of self-emulsification of the formulation [24]. Rate of emulsification is found to be dependent on nature of oil phase and oil/surfactant ratio [49]. Rapid rate of emulsification is observed with higher surfactant concentration because of rapid ejection of oil droplets by penetration of water into interface. The emulsification time can also be determined by visual evaluation after placing the formulation in 0.1 N HCl under stirring at body temperature by which the GI conditions can be simulated [41].

#### 6.4.5. Cloud Point Determination

Cloud point is generally determined by gradually increasing the temperature of water bath in which the formulation is placed and measured spectrophotometrically. The point where % transmittance decreases signifies the cloud point that is the temperature above which the transparent solution changes to cloudy solution. As the body temperature is 37°C, formulations should exhibit the cloud point more than body temperature to retain its self-emulsification property.

Phase separation and decrease in drug solubilization are commonly observed at higher temperature than the cloud point due to the susceptibility of surfactant to dehydration [42]. Cloud point is influenced by drug lipophilicity and other formulation components [24, 42].

#### 6.4.6. Viscosity Measurements

Viscosity of diluted SMEDDS formulation that is microemulsion is generally determined by rheometers like Brookfield cone and plate rheometer fitted with cone spindle [55] or rotating spindle Brookfield viscometer [56]. During titration, the initial increase in viscosity with subsequent decrease, with the increase in water volume attributed to water percolation threshold, indicates the formation of O/W microemulsion from W/O microemulsion with intermediate bicontinuous phase [57]. The rheology of microemulsion can be determined by the graph plotted between shear stress and shear rate. The Newtonian behavior indicates the presence of droplets of small and spherical shape [56].

#### 6.4.7. Dilution Studies

The effect of dilution on microemulsion clarity can be evaluated by performing the dilution of microemulsion preconcentrate to various dilutions that simulate the gastric conditions and in various diluents like double distilled water, simulated gastric fluid (SGF), and simulated intestinal fluid (SIF) [37]. If clarity is maintained on increased dilution and also in case of change in type of diluents, this indicates absence of drug precipitation [46]. The extent of dilution of SMEDDS to 100 times with all the above diluents can simulate the conditions* in vivo* [58]. Effect of pH of dilution medium can be investigated by the dilution of SMEDDS with different solvents like Buffer pH 1.2, Buffer pH 6.8, and so forth along with the distilled water and should be observed for transparency and efficiency of self-emulsification [38].

#### 6.4.8. Refractive Index

Refractive index is the property by which the isotropic nature of diluted SMEDDS that is microemulsion can be determined. Karamustafa and Ćelebi performed refractive index measurements of optimized formulation at 4°C and 25°C up to 6 hrs at different time intervals and concluded that there is no significant change in refractive index indicating the constant microemulsion structure [56]. The constant refractive index also indicates the thermodynamic stability of the formulation [53]. Usually the refractive index measurements are carried out using refractometers [57]. Refractive index is mainly dependent on two factors, that is, amount of the cosurfactant and globule size. Refractive index decreases with increase in cosurfactant concentration attributed to decrease in the rigidity of microemulsion structure and it increases with the increase in globule size [50].

#### 6.4.9. Percentage Transmittance

This test gives the indication of transparency of diluted SMEDDS formulation. It is determined spectrophotometrically after dilution of formulation with water, keeping water as blank. The percentage transmittance value near to 100% indicates clear and transparent microemulsion formation [40].

#### 6.4.10. Transmission Electron Microscopy (TEM) Study

It is mainly used to investigate the structure and morphology of microemulsions that are formed by dilution of SMEDDS [59]. These studies are performed by the combination of bright field imaging at increasing magnification and diffraction modes [60]. The diluted SMEDDS is placed on holey film grid and morphology can be determined [40, 46].

Basalious et al. [41] and Elnaggar et al. [42] performed TEM studies by staining the samples. In both experiments, the drop of diluted formulation was placed on copper grid and after staining with suitable stains like uranyl acetate it was dried and then the droplets were visualized for the detection of morphology like size and shape of the droplets. Some other stains like 1% phosphotungstic acid solution and 1% methylamine vanadate can also be used [52]. By TEM studies, the uniformity in droplet size can also be known [41].

#### 6.4.11. Differential Scanning Colorimetry

This is mainly used for the characterization of microemulsions that are formed by dilution of SMEDDS in terms of peaks corresponding to water. The peaks give information about the condition of water like bound state or free state [55]. Pure water is used as reference which shows large, sharp peak approximately at −17°C that indicates the freezing point. Podlogar et al. conducted DSC experiments on microemulsions of water-Tween 40/Imwitor 308-isopropyl myristate system and identified peaks corresponding to the water at lower temperature than the pure water (approximately at −45°C at 15% w/w) indicating the presence of water in the bound state in microemulsions preferably bound to surfactants. More increased concentration of water than this leads to the shift to higher temperature. From the observations of thermal behavior of water, they concluded that the high concentration of water (>35% W/W) produced O/W microemulsions [61].

#### 6.4.12. NMR Techniques

These are used to evaluate the structure of microemulsions formed after dilution of SMEDDS. The diffusive behavior of microemulsion components can be studied with the help of Fourier transforms pulsed gradient spin-echo method (PGSE). The transformations between microemulsion phase and bicontinuous phase upon dilution can be studied by PGSE-NMR method. By using 129xe NMR, the droplet size of the microemulsion can be determined by noting the shift of signal to higher field with respective increase in the size of the droplet [62]. The self-diffusion NMR studies are used to determine the type of microemulsion that is formed after dilution of SMEDDS and also to determine the transitions like W/O to bicontinuous and bicontinuous to O/W type upon incremental dilution. In this technique, the self-diffusion coefficients of different components of microemulsion are compared with that of pure components. If the diffusion of one of the components is lower than that of pure component, this indicates the presence of droplets, that is, O/W or W/O, and usually surfactant and cosurfactants also have slow diffusion because of the formation of film around the droplets by these components. If oil and aqueous phase have high diffusion coefficients and are of the same magnitude as pure components, it indicates the presence of bicontinuous type microemulsion [55].

#### 6.4.13. Small Angle X-Ray and Neutron Scattering Methods

Small angle X-ray scattering techniques are useful for the characterization of structures that are formed by dilution of SMEDDS. Evaluation of liquid crystalline structures formed by the dilution of SMEDDS is important as these govern the stability of formulation, self-emulsification, and extent of drug release. Goddeeris et al. performed small angle X-ray scattering studies on formulations containing different proportions of water. At 10% w/w (lower) water concentration, random periodic or lamellar structure was observed and at 20% w/w water concentration, lamellar structures were observed. Further increase in water concentration to 40% w/w revealed hexagonal or lamellar structures. The temperature increase to 37°C from 25°C did not cause significant changes in liquid crystalline structures that are formed [63]. Small angle neutron scattering methods are useful to determine transitions in microemulsion structures upon dilution and also to determine size and shape of the droplets [30, 62].

#### 6.4.14. Thermodynamic Stability Studies

These studies are useful to evaluate the consequence of temperature change on formulation. Formulation is diluted with aqueous phase and subjected to centrifugation at 15,000 rpm for 15 min [46] or at 3500 rpm for 30 min [59]. The samples in which the phase separation is not observed are subjected to freeze thaw cycles (−20°C and 40°C temperature, resp.) and observed visually. The thermodynamically stable formulations will not show any change in visual description [46, 59].

#### 6.4.15. *In Vitro* Dissolution Profile

Drug release from formulation can be evaluated after filling the formulation in a hard gelatin capsule using USP XXIII apparatus I at 100 rpm [44, 64, 65] or USPXXIII apparatus II at 50 rpm [40, 49] or with dialysis method [66] at 37 ± 0.5°C. Samples at regular intervals should be withdrawn from the medium and drug content is estimated and compared with the control. The polarity of oil droplet has impact on drug release from the diluted SMEDDS. The higher the polarity, the faster the drug release from the oil droplet into the aqueous phase. Polarity is mainly dependent on the HLB of surfactant, molecular weight of hydrophilic part of the surfactant, and its concentration along with the degree of unsaturation of fatty acid of lipid phase [3, 18].

In a study performed by Jantratid et al., comparison is made between the drug release profile using paddle type apparatus and that of reciprocating cylinder and it was found that the use of USP apparatus 3 (reciprocating cylinder, Bio-Dis) for the evaluation of drug release from the liquid lipid dosage forms like SMEDDS is more suitable than the paddle method and produced reproducible results compared to the paddle method and concluded that this type of behavior is attributed to the uniform break-up of oil layer by the movement of inner cylinder with mesh inserts compared to the paddle method [67].

#### 6.4.16. Stability Assessment

Stability studies are performed as per the ICH guidelines on the formulation which is filled in gelatin capsules. At regular intervals the samples should be collected and tested for appearance, color, drug content, pH of diluted formulation, and dissolution profile. If there is no change in all these properties during storage conditions, formulation can be concluded as stable formulation [38, 46, 54, 65].

## 7. Conclusion

Self-microemulsifying drug delivery systems are recent and effective approach for the augmentation of oral bioavailability of many poor water soluble drugs provided that the drug should be potent with high lipid solubility. It is well demonstrated that SMEDDS promotes lymphatic delivery of extremely hydrophobic drugs (with high octanol : water partition coefficient) with good solubility (>50 mg/mL) in triglycerides. Faster and enhanced drug release can be attained with smaller droplets which in turn promotes bioavailability. The present review highlighted the developmental steps (solubility studies, construction of pseudoternary phase diagrams, and various evaluation tests) involved in obtaining a robust and stable dosage form. Further research in developing SMEDDS with surfactants of low toxicity and to develop* in vitro *methods to better understand the* in vivo* fate of these formulations can maximize the availability of SMEEDS in market.

## Figures and Tables

**Figure 1:**
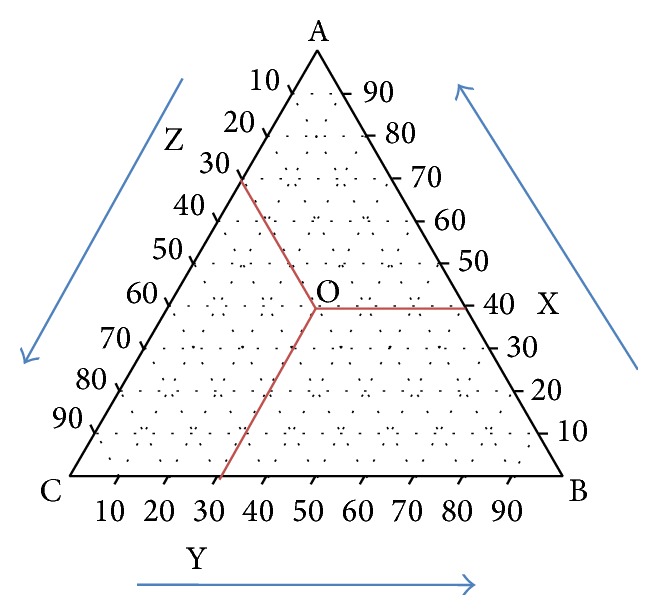
Typical ternary diagram indicating the composition of A, B, and C at point O.
